# A modified root reinforcement technique for acute aortic dissection with a weakened aortic root: a modified Florida sleeve technique and two cases report

**DOI:** 10.1186/1749-8090-8-203

**Published:** 2013-10-31

**Authors:** Woon Heo, Ho-Ki Min, Do Kyun Kang, Hee Jae Jun, Youn-Ho Hwang, Jin Ho Choi, Jin Hong Wi

**Affiliations:** 1 Department of Thoracic and Cardiovascular Surgery, Haeundae Paik Hospital, Inje University College of Medicine, 875 (Jwadong) Haeundae-ro, Haeundaegu, Busan 612-030, Korea; 2 Department of Thoracic and Cardiovascular Surgery, Eulji University Hospital, Eulji University School of Medicine, Daejeon, Korea; 3 Department of Thoracic and Cardiovascular Surgery, Severance Cardiovascular Hospital, Yonsei University College of Medicine, Seoul, Korea

**Keywords:** Dissection, Aortic root, Blood vessel prosthesis

## Abstract

Despite marvelous advances in repair for acute type A aortic dissection over past decades, it remains challenging to repair the aortic root when aortic dissection extended to the sinuses causes the fragile root because of its thinner layers, which are susceptible to suture trauma. Here, we describe a modified Florida sleeve technique to strengthen the weakened aortic root. After mobilization of the aortic root and the coronary arteries, a designed Dacron tube graft was wrapped outside the sinuses as neo-adventitia to reinforce the dissected weakened wall. During surgery for aortic dissection, our technique is easy and effective to reinforce a weakened root and avoid bleeding. Furthermore, this might be an alternative technique to restore and maintain the geometry of the aortic root.

## Background

Acute type A aortic dissection remains a difficult to treat and catastrophic disease. There have been many therapeutic advances over past decades to improve treatment outcomes. However, the repair of the fragile aortic root still remains issuable and controversial for the surgical correction of acute aortic dissection. Aortic dissection widely extended to the sinuses might to leave a thinner outer layer, which is susceptible to suture trauma and surgical bleeding. In this situation, even a slightly misplaced stitch may cause disrupted suture holes, which may lead to intractable hemorrhage arising from deep and long suture lines because of the fragility of tissue. To overcome bleeding and strengthen the dissecting flaps, multiple surgical options were advocated including reinforcement with Teflon felts, glue fixation, partial aortic root remodeling, neomedia formation, and a patch neointima formation [1-6]. We modify a Florida sleeve technique into a new technique, named as a neo-adventitia technique, by wrapping outside the sinus with a cylindrical Dacron graft to overcome bleeding and reinforce a dissected aortic root.

## Case presentation

Two Korean patients with acute type A aortic dissection were performed operations using our modified technique, and the medical records were reviewed retrospectively. Patient 1, a 78-year-old woman, had a 10 year history of hypertension and her preoperative echocardiogram revealed preserved left ventricular function, trivial aortic regurgitation, and moderate amount of pericardial effusion. Patient 2, a 72-year-old man, had an unremarkable past history and his preoperative echocardiogram noted preserved left ventricular function and moderate to severe aortic stenosis with a bicuspid aortic valve. Chest CT confirmed acute type A aortic dissection for each patient, and emergent surgeries were performed.

### Operative technique>

Cardiopulmonary bypass (CPB) was instituted with cannulations in the right axillary and femoral arteries and in the right atrium and systemic cooling was started. Under cardioplegic arrest, the ascending aorta was transected just above the sinotubular junction, and the intraaortic pathology was examined. Our indications for reconstruction of the aortic root with a modified Florida sleeve technique are a deeply dissected sinus as far as the level of the aortic annulus or a weakened aortic root.

**Figure 1:**
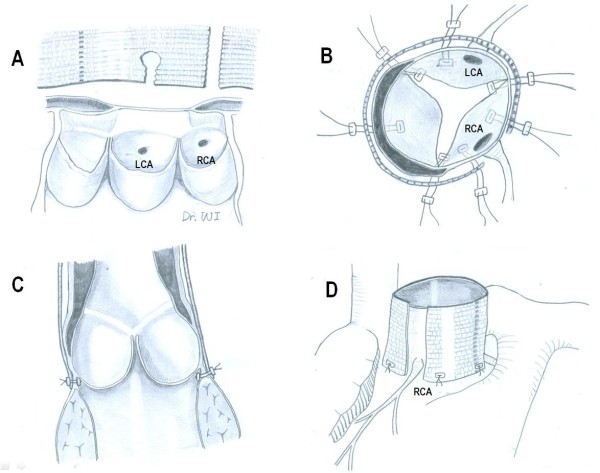
**Schematic diagram of the operation. (A)** A vertical slit of the graft corresponding to the estimated distance from the aortoventricular junction to the bottom of the left main trunk and a round opening were created from the bottom to midpoint. And the graft was cut longitudinally over the entire length at the marked position corresponding to the right coronary artery. **(B)** A wrapping graft as "neo-adventitia" is seated down after multiple subannular inside-out sutures of 4–0 polypropylene sutures with Teflon pledgets were placed in a horizontal mattress fashion. **(C and D)** Transsectional and external diagrams show after completion. LCA: left coronary artery; RCA: right coronary artery.

The aortic root was completely mobilized circumferentially down to the aortic annular level, including mobilization of the proximal portions of the coronary arteries. Exposures under the coronary arteries weren’t performed to avoid bleeding, iatrogenic injury, and time consuming. Seven to eight subannular inside-out sutures of Teflon pledget reinforced 4–0 polypropylene were placed in a horizontal mattress fashion just below the aortic annulus; 3 were in line with the commissure and the others were below the midpoint of each leaflet (Figure 1A). The locations of the coronary arteries were marked on the graft, which was cut off about 5 cm long from a cylindrical graft (Vascutek Ltd, Renfrewshire, UK) selected for ascending aorta replacement by sizing the sino-tubular junction and the distal ascending aorta. A distance from the aortoventricular junction where the sutures had exited to the bottom of the left coronary trunk was estimated. A vertical slit of the graft corresponding to the estimated distance from the aortic annulus to the bottom of the left main trunk and a round opening were created by using an ophthalmic electrocautery from the bottom to midpoint. And the graft was cut longitudinally over the entire length at the marked position corresponding to the right coronary artery (Figure 1A). At first, the 2 subannular sutures on each side of the left main trunk were passed through the base of the graft, and the graft was seated over the root by carefully aligning the "round opening" for the left main trunk. The other subannular sutures were passed through the base of the graft with considering appropriate position and tied down reinforced with counter-pledgets. But the slit in the graft below the left main trunk were left open and the slit for the right coronary arteries remained fully open without reconnection. In patient 1, she underwent aortic valve-sparing procedure because of good valve morphology. Resuspension of each aortic commissure was performed using 5–0 polypropylene sutures with Teflon pledgets placed on both the inner and outer sides of the root with considering that proper alignment and elevation of the commissures might be important to ensure valvular competency. The leaflets were inspected and confirmed for coaptation. Patient 2 revealed a bicuspid aortic valve with moderate to severe aortic stenosis, so the aortic valve was replaced with a tissue valve. Under circulatory arrest with selective cerebral perfusion, the aortic clamp was removed and the dissected aortic tissue that contained an intimal tear was resected, and then distal graft-to-aortic anastomosis was completed. After deairing and clamping of graft, CPB was re-initiated and rewarming was started. After the sleeve of a wrapping graft and the native aorta were cut off at the same level of the sinotubular junction, proximal graft-to-root anastomosis was completed with considering that full thickness suturing, together with the dissected aortic wall and a wrapping graft, is important for unifying the layers of the dissected aortic root. And we reinforced the proximal suture line with using multiple 4–0 polypropylene sutures with Teflon pledgets, but didn’t use Teflon felt strip or biologic glue.

**Figure 2:**
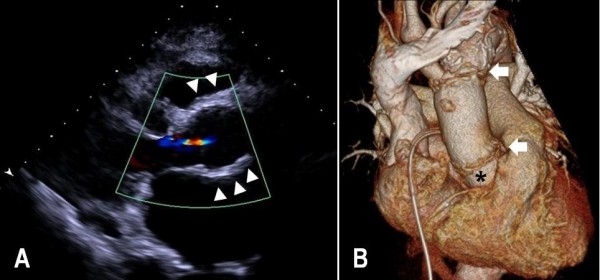
**Post-operative images. (A)** Post-operative transthoracic echocardiographic image with color flow mapping from the parasternal long-axis vies in patient 1. The geometry of the aortic root reinforced by wrapping a Dacron graft (Arrowheads) is well restored and maintained as a bulging morphology. And well-coaptating aortic valve cusps with trivial aortic regurgitation are also noted. **(B)** Post-operative three-dimensional volume rendering of contrast-enhanced CT scans in patient 1. It reveals that the ascending aorta was replaced with a prosthetic graft and the aortic root was reinforced by wrapping a Dacron graft (asterisk). Also proximal and distal anastomotic lines (arrow) were visible.

The postoperative course was uneventful and the chest tube drainages of the first 24 hours were 640 ml and 280 ml respectively. Post-operative echocardiogram revealed trivial aortic regurgitation in patient 1 (Figure 2A) and a well-functioning aortic prosthesis in patient 2. They remain asymptomatic for 3 months and 2 months respectively. Post-operative follow-up chest CT scans were performed and revealed the patent bypass grafts (Figure 2B).

## Discussion

Our technique is summarized as followed; 1 > wrapping with a Dacron tube graft outside the aortic root (not total, but nearly total) to reinforce the dissected weakened wall, 2 > no resection of any sinuses of Valsalva, and 3 > for aortic valve sparing procedure in patient 1, resuspension of each commissure to the outer graft to restore the normal root geometry and maintain a coaptation area above an aortic annuls level. This is motivated by valve reimplantation technique and wrapping techniques of the aortic root with a vascular graft [5,7-11]. We named this technique a modified Florida sleeve or "neo-adventitia technique". When the aortic root is widely dissected, our indications for the aortic root reconstruction are as followed; 1 > if there is the structurally abnormal or calcified aortic valve with an irreparable or ectatic aortic root, composite replacement is underwent. 2 > if the structurally abnormal or calcified aortic valve without root pathology, aortic valve replacement combined with a root reinforcement using our new technique are underwent, 3 > if normal valve morphology with an ectatic root (over 50 to 55 mm in diameter or over 45 to 50 mm in diameter if a younger patient or a patient with connective tissue disease), a valve-sparing reimplantation or remodeling is performed, and 4 > if normal valve with normal or mild ectatic root (under 45 mm in diameter), our new technique is used.

In our technique, there are several advantages compared with other root replacement or valve sparing procedure.

First, it is probably the easier and quicker approach. Root replacement with composite grafts or valve-sparing technique has been applied with good results to treat a widely dissected or irreparable aortic root. However, it implies prolonged surgical duration and technically demanding because the native aortic valve or coronary ostia needs to be reimplanted. Its properties might confer additional risk upon unstable patients under emergency conditions and drive to be needed a faster and simpler procedure. Our technique may be an alternative approach. Even in the second case, we believe that our modified technique with separate ascending aorta-aortic valve replacement are easier and quicker approach than root replacement with composite graft.

Second, this can avoid uncontrollable bleeding from a proximal anastomotic line. When a weakened aortic root exists, the main mechanism of bleeding may arise from further damage to the fragile root wall from the suture line such as suture-hole avulsions. Furthermore, even a slightly misplaced stitch may cause disrupted suture holes, which may lead to intractable hemorrhage arising from deep and long suture lines because of the fragility of tissue. Our technique can avoid to suture through a weakened root wall by accomplishing with 2 suture lines (one through the plane below the aortic valve and another at the level of the sinotubular junction) and minimize externally exposed suture lines compared with the Urbanski technique or root remodeling [6,12]. Moreover, outer graft as a neo-adventitia is effective to seal suture holes, even if avulsion.

Third, it can prevent a future root dilatation, which may be favorable to the preserved valve function. Supracommissural graft replacement is frequently selected because of technical simplicity and less invasiveness. However, patients who undergo supracommissural graft replacement are at a risk of requiring reoperation due to a variety of complications, including sinus of Valsalva dilatation, progressive aortic regurgitation, redissection, or residual dissection at the aortic root [3]. Development of postoperative aortic regurgitation is a significant cause for reoperation after supracommissural graft replacement. Aortic regurgitation can recur due to redissection or development of aortic root dilatation during the midterm period [3]. There may be a concern for a future root dilatation around neo-adventitia defect. However, it would be diminished by the fact that the most frequently affected sinus of Valsalva is the non-coronary sinus, which was fully covered.

Lastly, it might be less stressful to a surgeon for sizing a graft because of a full vertical slit of a graft made for the right coronary, which was left open without reconnection after seating a graft. Neo-adventitia defect around the right coronary artery is in the superficial and anterior surgical field. Once bleeding occurs after the anastomosis, hemostasis in this anterior and superficial field is easier compared with the area around the left main trunk.

## Conclusions

During surgery for acute type A aortic dissection, our technique is an easy and effective way to reinforce a weakened root and avoid bleeding. Furthermore, this might be an alternative technique to restore and maintain the geometry of the aortic root for valve-sparing procedure for selected patients with acute type A aortic dissection.

## Consent

Written informed consent was obtained from the patient for publication of this case report and all accompanying images. A copy of the written consent is available for review by the Editor-in-Chief of this journal.

## Abbreviations

CT: Computed tomography; CPB: Cardiopulmonary bypass; RCA: Right coronary artery; LCA: Left coronary artery.

## Competing interests

The authors declare that they have no competing interests.

## Authors’ contributions

WH: participated in the management and wrote the manuscript. HM: performed surgery on the patient and revised the manuscript. DK: revised the manuscript. HJ: revised the manuscript. YH: revised the manuscript. JC: revised the manuscript. All authors read and approved the final manuscript.

## Authors’ information

HM - Is a surgeon of the Thoracic and Cardiovascular Surgery department at the Haeundae Paik Hospital where the patient underwent operation. He is also an assistant professor at the Inje University College of Medicine in Busan, Korea.
